# Engineering of an Fc-specific monovalent protein G for the light-controlled affinity purification of antibodies

**DOI:** 10.1038/s41598-025-25894-5

**Published:** 2025-10-31

**Authors:** Peter Mayrhofer, Arne Skerra

**Affiliations:** Chair of Biological Chemistry, School of Life Sciences, Technical University of Munich, 85354 Freising, Germany

**Keywords:** Blood proteins, Antibody therapy, Protein aggregation, Protein purification, Chromatography

## Abstract

Like other widely applied bacterial surface receptor proteins for immunoglobulins (Igs), such as protein A and protein L, the Ig-binding domain of protein G (ProtG) has dual binding activity. ProtG can independently associate both with the Fc region of an antibody (mAb) and with its Fab and, thus, provoke cross-linking if applied in solution. Indeed, we observed pronounced precipitation activity when using ProtG equipped with the Azo-tag as a small adapter molecule for the light-controlled affinity purification of mAbs. We demonstrate that this undesired precipitation phenomenon follows the classical Heidelberger-Kendall curve. Furthermore, we describe a mutant of ProtG in which Asn478 at the interface with the Fab is replaced by Arg, which results in the effective loss of this secondary binding activity while maintaining high affinity towards the Ig Fc region. ProtG^N478R^ no longer induces precipitation when mixed with a series of medically relevant mAbs. Hence, Azo-ProtG^N478R^ can be applied as a convenient molecular tool to isolate antibodies from cell culture medium—even with a high content of albumin—in a single step via Excitography. In this technique, elution is triggered by *trans* → *cis* isomerisation of the Azo-tag upon illumination with mild UV-A light and a harsh pH shift is avoided.

**Supplementary Information:**

The online version contains supplementary material available at 10.1038/s41598-025-25894-5.

## Introduction

Bacterial immunoglobulin (Ig)-binding proteins, in particular protein A from *Staphylococcus aureus*^1^ and protein G from group G *Streptococci*^2^, are widely used for the affinity purification of recombinant or monoclonal antibodies (mAbs) both in biomedical research and in the industrial manufacturing of Ig-based biopharmaceuticals^3,4^. Various robust chromatography resins carrying different covalently immobilized versions of protein A or G are commercially available and allow the rapid isolation of antibodies or Fc-fusion proteins from conditioned cell culture supernatants under standardized conditions. However, one caveat is the use of acid elution buffers to dissociate the protein–protein complex and recover the Ig from the affinity column. Such low pH conditions can promote both protein aggregation and deamidation of Asn and Gln side chains^5,6^, which is a concern not only for biopharmaceutical manufacturing but also for those areas of fundamental research where homogenous and fully functional protein preparations are crucial.

We have recently developed the Azo-tag, which comprises a *cis*/*trans*-isomerizable azobenzene side chain, for the light-controlled affinity chromatography—dubbed Excitography—of a wide range of proteins, including antibodies, under native buffer conditions^7^. In this technique, the non-canonical amino acid p-(phenylazo)-L-phenylalanine (Pap) is cotranslationally incorporated into the recombinant protein, as part of the short, 3–4 residue Azo-tag, via amber stop codon suppression using an expanded genetic code. The light-dependent *trans* → *cis* isomerization of the Pap side chain is utilized to adsorb and desorb the protein to/from an α-cyclodextrin (α-CD) affinity matrix under different illumination conditions, thus allowing elution simply under mild UV-A radiation in a physiological buffer of choice, directly suitable for subsequent biochemical or cell culture assays.

When applying Excitography to mAbs, instead of incorporating the Azo-tag at the genetic level into the Ig protein a tiny adapter molecule was employed. To this end, the small soluble Ig-binding C2 domain of protein G—a 56-residues fragment of the natural multi-domain protein anchored in the bacterial membrane^2^—was equipped with an N-terminal Azo-tag and dubbed Azo-ProtG^7^. When adding Azo-ProtG, produced in an *E. coli* expression system, to a cell culture medium containing the mAb, the antibody was affinity-purified via Excitography in one step and in a highly efficient manner using a native buffer. However, during this application of ProtG as affinity adapter molecule in solution we observed that the mixing ratio with the antibody was critical to prevent the formation of a precipitate. Interestingly, it turned out that this Ig precipitation behavior upon addition of ProtG, i.e. a single Ig-binding domain of the larger protein G, was reminiscent of the long-known Heidelberger-Kendall curve.

In a series of seminal publications, Heidelberger & Kendall^8,9^ described the quantitative principles of the so-called precipitin reaction, which refers to an antibody forming a precipitate from a mixed solution with its antigen due to multivalent non-covalent complex formation. The resulting precipitin curve describes the relationship between the concentration of the antigen and the amount of precipitate formed in the presence of a constant quantity of antibody, comprising three characteristic zones^10^: (i) in the zone of antibody excess, there is only minor precipitate formation as all binding sites on the antigen are saturated; (ii) in the so-called zone of equivalence, precipitation is maximal owing to multiple intermolecular cross-links between antibodies and antigens upon binding (also known as immune complex); (iii) in the region of high antigen concentration, precipitation is low again as all binding sites of the antibody become saturated.

**Fig. 1:**
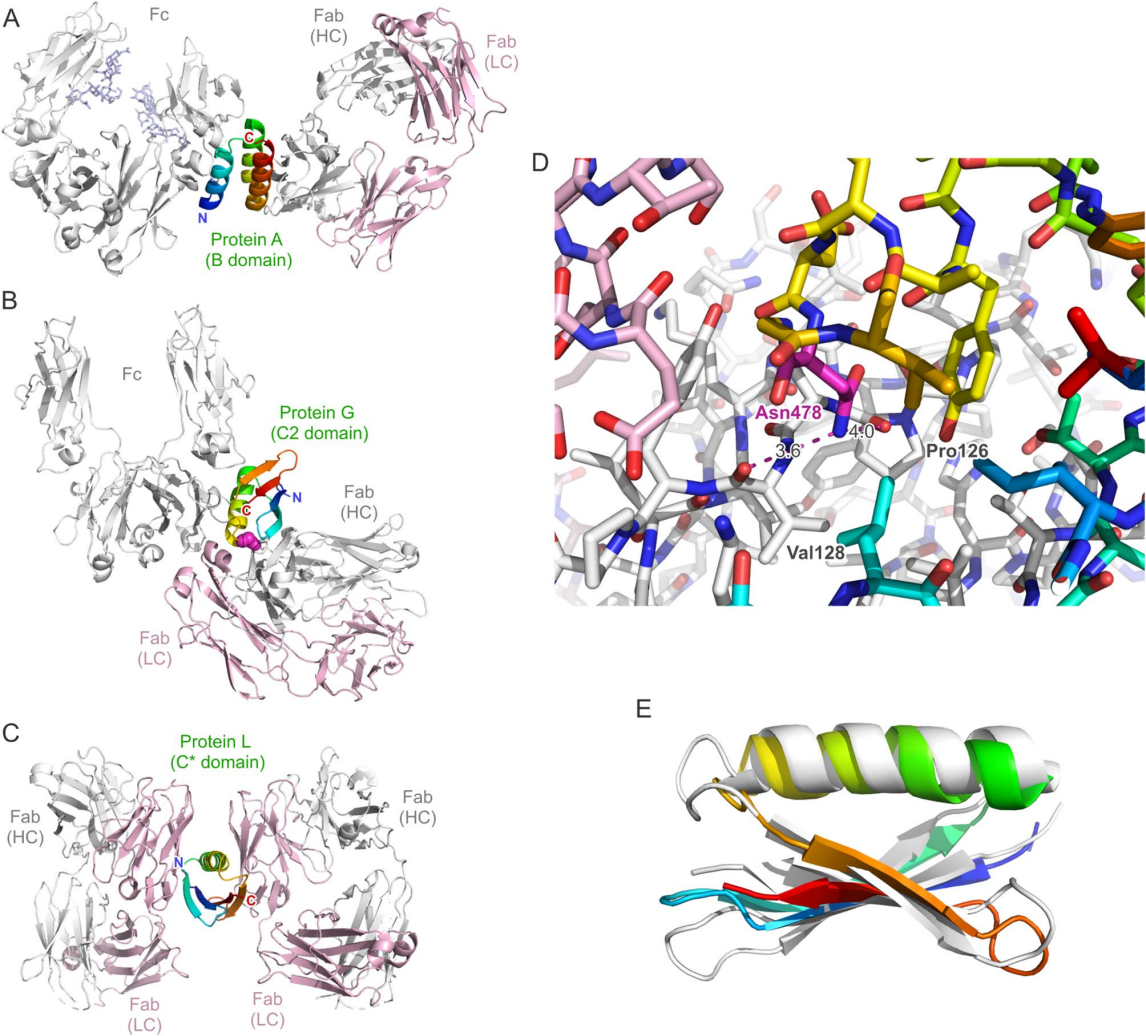
(**A**-**C**) Dual complexes between antibody Fc and Fab fragments and individual Ig-binding domains of the cell wall-anchored surface receptors protein A, protein G and protein L from Gram-positive bacteria based on crystallographic analyses. (**A**) The B domain of protein A in complex with an IgG1 Fc fragment (PDB ID: 5U4Y) (sugar side chains in the C_H_2 domain included) superimposed onto its complex with the trastuzumab Fab fragment (PDB ID: 4HKZ). (**B**) The C2 fragment of protein G in complex with an IgG1 Fc fragment (PDB ID: 1FCC) superimposed onto its complex (here the domain III of protein G from *Streptococcus* sp. G148) with the MOPC21 Fab fragment (PDB ID: 1IGC). (**C**) The C* domain of protein L simultaneously in complex with two copies of the same human IgM/κ 2A2 Fab fragment (PDB ID: 1HEZ). All protein models are shown in cartoon style, with the Ig light chain colored pink and the heavy chain colored light grey. The bacterial Ig-binding domain is colored rainbow (amino- to carboxy-terminus: blue to red), and residue Asn478 in ProtG (B) is highlighted as magenta spheres. (**D**) Close-up view of the interface region between ProtG (carbon atoms rainbow-colored) and a Fab (carbon atoms pink or light grey for the light and heavy chains, respectively) around its residue Asn478 (PDB ID: 1IGC; note that the Asn residue is numbered 42 in this coordinate set). The carboxamide group in the side chain of this Asn residue has been flipped by 180° to yield two chemically plausible hydrogen bonds with the carbonyl oxygens of Pro126 and Val128 in the Fab heavy chain (indicated by magenta broken lines). (**E**) Superposition between ProtG (rainbow-colored) and the C* domain of protein L (PDB ID: 1HEZ; light grey) to illustrate their related protein fold (RMSD = 2.56 Å for 54 paired Cα atoms).

**Fig. 2:**
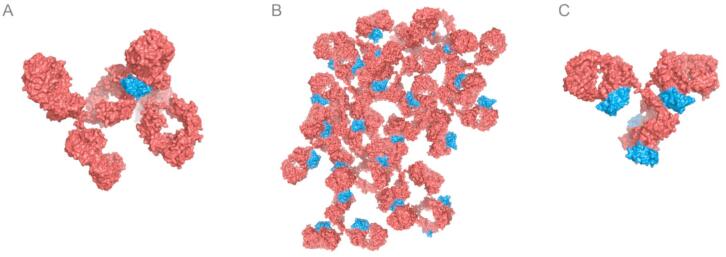
Illustration of a multimeric “immune complex” formed between the bivalent/bispecific C2 domain of protein G (shown as a blue surface representation) and a typical IgG antibody (red) mediated via its Fab and Fc portions, depending on the stoichiometric ratio. (**A**) The Ig-binding domain saturated with two antibody molecules (if the antibody is present in excess); (**B**) schematic depiction of a multimeric immune complex between ProtG and mAb (at conditions of equivalent concentrations); (**C**) the antibody saturated with four Ig-binding domains (if protein G is present in excess). The molecular models were generated using PyMOL graphics software (Schrödinger, New York, NY) based on suitable crystal structures (PDB IDs: 1IGT, 1FCC, 1IGC).

While this general relationship was originally uncovered in experiments with polyclonal sera and macromolecular antigens displaying multiple epitopes, the same fundamental effect can occur with immunochemical components having less complex composition, such as purified bivalent or bispecific mAbs and oligomeric or oligovalent protein antigens. In this regard, it is noteworthy that a single Ig-binding domain of protein G exhibits two distinct and independent Ig-binding sites^11,12^: one specific for the Fc portion of IgG and one specific for the Fab—albeit with lower affinity^13^. Due to the circumstance that the two corresponding interfaces on the ProtG domain do not overlap (Fig. 1), thus allowing binding of two antibody molecules simultaneously, a polymeric complex may be formed when ProtG is mixed with a mAb at suitable ratio (Fig. 2).

Based on the hypothesis that the secondary, Fab-specific binding site of ProtG may effect the crosslinking of antibody molecules (regardless of their antigen specificities) and, thus, lead to the formation of macromolecular immune complexes, we have investigated this phenomenon in greater detail. We report a mutagenesis study to eliminate the binding activity towards the Fab region of antibodies and the development of a truly monovalent Fc-specific ProtG version with improved applicability, in particular with regard to the light-controlled affinity purification of mAbs.

## Results

An ideal Ig-binding domain—and viable Azo-ProtG adapter protein—would lack a secondary binding activity towards a Fab while preserving high affinity towards the Fc portion of IgG. According to the crystallized complex between the C2 fragment of *streptococcal* protein G in complex with an IgG1 Fc fragment^12^, residue Asn478 (numbering according to UniProt ID: P19909, which corresponds to the longer protein G version of strain G148; annotated as Asn37 in PDB ID: 1FCC and as Asn42 in PDB ID: 1IGC) does not participate in the interaction with Fc. Its side chain is positioned at the C-terminal end of the α-helix which packs against a four-stranded β-sheet within the Ig-binding domain (Fig. 1B). On the other hand, in the complex with the Fab of the antibody MOPC21^11^ Asn478 is buried at the interface with the C_H_1 domain (Fig. 1D). There, its side chain carboxamide group forms weak hydrogen bonds with the two carbonyl oxygens of residues Pro126 and Val128 in the Ig heavy chain fragment of the Fab (numbering according to PDB ID: 1IGC).

Hence, this position appeared suitable to introduce different side chains that are incompatible with this kind of complex formation. To this end, Asn478 was replaced by Glu, Lys and Arg. Among these, the Arg residue with its longer and much more voluminous side chain, which exhibits extended rotational degrees of freedom and carries an additional charge with strong solvation potential, appeared particularly promising. In fact, this mutant also showed the highest yield among the mutations tested, comparable with the wild-type ProtG domain, using our expression system for Azo-tagged proteins in *E. coli*^7^. Moreover, measurement of its thermal unfolding stability using circular dichroism (CD) spectroscopy revealed a slightly higher stability, with T_M_ = 79.8 °C *versus* 78.7 °C, for this mutant (Supplementary Fig. S1).

**Fig. 3:**
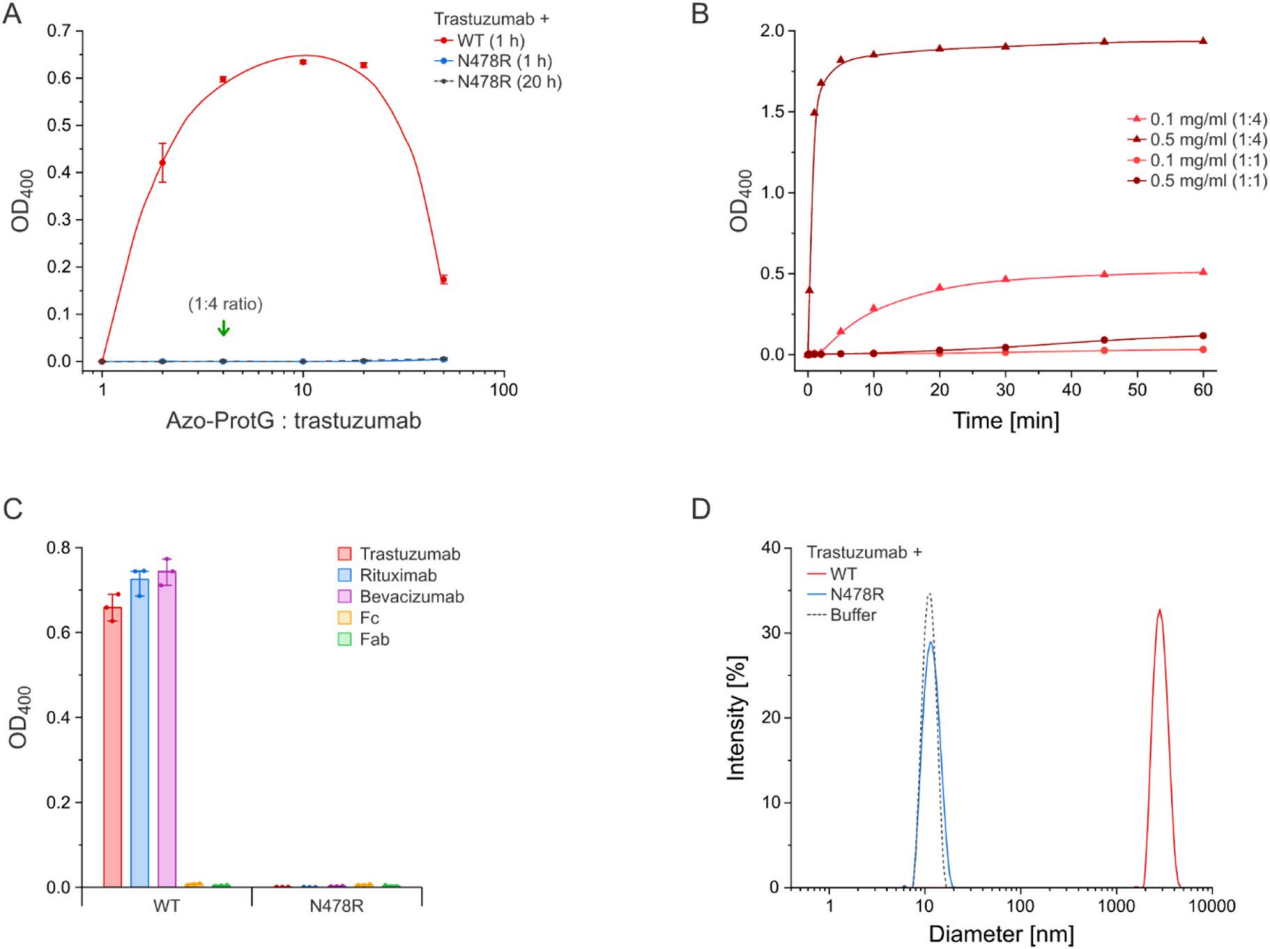
Effect of the Asn478 → Arg substitution in the C2 fragment of protein G (ProtG) on its capacity to trigger antibody precipitation. (**A**) Incubation of a mAb with ProtG leads to a precipitin reaction in accordance with the Heidelberger-Kendall curve. Trastuzumab (0.1 mg/ml) was incubated with wild-type Azo-ProtG (1:1–1:50 molar ratio) at room temperature for 1 h, or with Azo-ProtG^N478R^ for 1 h or 20 h, and the OD_400_ was measured as an indicator of turbidity (N = 3). The green arrow marks the stoichiometric ratio with respect to the four binding sites for wild-type ProtG per full-size mAb molecule. (**B**) Kinetics of the precipitin reaction with ProtG. 0.1 mg/ml or 0.5 mg/ml trastuzumab was each incubated with wild-type Azo-ProtG at a 1:1 or 1:4 molar ratio and the OD_400_ was monitored. (**C**) Three different medically relevant antibodies, trastuzumab, bevacizumab and rituximab, an Fc fragment, and the Fab fragment of trastuzumab were incubated (each at 0.1 mg/ml) with wild-type Azo-ProtG or Azo-ProtG^N478R^ (1:4 molar ratio) for 20 h at room temperature and the OD_400_ was measured (N = 3). (**D**) Investigation of the protein aggregate size by DLS. Trastuzumab (0.5 mg/ml) was incubated with wild-type Azo-ProtG or Azo-ProtG^N478R^ at a 1:4 molar ratio, or just using buffer, for 20 h at room temperature prior to the measurement. Lines connecting the data points in (A) and (B) correspond to spline interpolation.

The functional effect of the Asn478 → Arg mutation in ProtG was initially investigated with regard to the formation of precipitate upon mixing with an antibody by using a turbidity assay (Fig. 3). In this kind of experiment the increase in optical density (OD) due to the scattering of aggregated protein particles is measured in a UV/Vis spectrophotometer at wavelengths between 340 and 400 nm^14–17^. Due to the peak absorption of the Azo-tag at 335 nm in connection with its *trans*-to-*cis* isomerization^7^, OD measurements were here performed at the longer wavelength of 400 nm.

For the turbidity assay, varying amounts of the purified Azo-ProtG, both wild-type (wt) and the Asn478 → Arg mutant, were mixed with a well known antibody, trastuzumab (at a final concentration of 0.1 mg/ml), to yield molar ratios of 1:1 to 50:1 (relative to the mAb). After incubation at room temperature for 1 h, the OD_400_ value was measured (Fig. 3A). At a 1:1 molar ratio, no significant increase in optical density or visible aggregate formation was detected for the ordinary Azo-ProtG. However, precipitation was observed already at a ratio of 2:1 and was almost maximal at 4:1 (i.e. the stoichiometric ratio to saturate all four possible wtProtG binding sites per mAb). Conversely, a considerably higher excess of Azo-ProtG (50:1) led to significantly less protein aggregation. Thus, the resulting bell-shaped precipitation curve resembled the classical relationship observed for the antibody-antigen precipitin reaction according to Heidelberger & Kendall^10^.

Of note, aggregation was relatively rapid in the presence of a moderate excess of Azo-ProtG (Fig. 3B). A 0.1 mg/ml mAb solution mixed with Azo-ProtG at a 1:4 molar ratio reached essentially maximal turbidity after 30 min. However, the kinetics of the reaction was strongly influenced by the protein concentration, as for a 0.5 mg/ml antibody solution mixed with Azo-ProtG at the same molar ratio precipitation was almost complete already after 5 min. Even a low 1:1 molar ratio resulted in partial aggregation at this higher antibody concentration, although with a longer lag phase of 10 min before a significant increase in turbidity became evident. Since considerably higher concentrations of recombinant mAbs are typically achieved in cell culture supernatants, wtProtG appeared unsuitable for preparative complex formation in solution and, in particular, for the light-controlled purification technique.

In contrast, after exchange of Asn478 by Arg (Azo-ProtG^N478R^), no increase in OD_400_ was observed in the same experimental setup over the whole range of tested ratios with the mAb, even after extended incubation for 20 h at room temperature (Fig. 3A). This effect was not limited to trastuzumab, as precipitation was equally absent for a different humanized antibody, bevacizumab, and for a mouse/human chimeric antibody, rituximab, whereas strong aggregate formation was observed in the presence of Azo-wtProtG for all three mAbs (Fig. 3C). Interestingly, in line with our cross-linking hypothesis, Azo-wtProtG did not trigger the precipitation of the isolated IgG1 Fc fragment—which was prepared by IdeS digest of bevacizumab (see Methods)—or of the recombinant Fab fragment of trastuzumab.

The macromolecular properties of the protein aggregates formed between ProtG and antibodies were investigated by dynamic light scattering (DLS), a method that is independent of the OD measurements performed so far. When incubating trastuzumab (0.5 mg/ml final concentration) with Azo-ProtG^N478R^ at a 1:4 molar ratio for 20 h at room temperature, the size distribution resembled that of the plain antibody (Fig. 3D). Just a minor shift of the unique peak in the particle size distribution was observed, from 10.8 nm Z-average diameter for the mAb alone to 11.2 nm in the presence of Azo-ProtG^N478R^, in line with the formation of a monodisperse antibody-ProtG complex. In contrast, only very large particles, with a Z-average diameter of 3.2 µm, were detected when the mAb was incubated with Azo-wtProtG under the same conditions.

These results indicate a substantial change in the Ig-binding properties of ProtG due to the single Asn478 → Arg substitution. To determine in greater detail how this mutation affects the interaction of the protein G domain with the Fc and Fab portions of an antibody, affinities were determined via real-time surface plasmon resonance (SPR) measurements (Table 1**, **Supplementary Fig. S2). When using the recombinant trastuzumab Fab^18^ as analyte a substantial decrease in affinity of the mutated Azo-ProtG^N478R^ became evident. While a K_D_ value of approximately 20 µM was determined for Azo-wtProtG, no significant response could be measured with the mutated protein at sample concentrations up to 256 µM, suggesting a loss in affinity for this interaction by several orders of magnitude.

**Table 1:** Mean K_D_ values as determined by SPR measurements (N = 3) using a steady-state fit.

	Trastuzumab	IgG1 Fc	Trastuzumab Fab
Azo-ProtG	564 ± 10 nM	499 ± 35 nM	19.7 ± 0.5 µM
Azo-ProtG^N478R^	459 ± 8 nM	682 ± 33 nM	$\gg$ 256 µM

In contrast, the replacement of Asn478 by Arg did not seem to play a significant role in the interaction with the Fc fragment, as very similar K_D_ values of ~ 500 nM *versus* ~ 680 nM were measured for the wild-type Azo-ProtG and its mutant, respectively, when using the proteolytically prepared IgG1 Fc fragment as analyte. In similar measurements with the intact trastuzumab, the dissociation constants for both versions of ProtG were even more similar (Table 1)—and also in close agreement with published values for the affinity between the unmodified C2 domain of protein G and intact human IgG^19^.

**Fig. 4:**
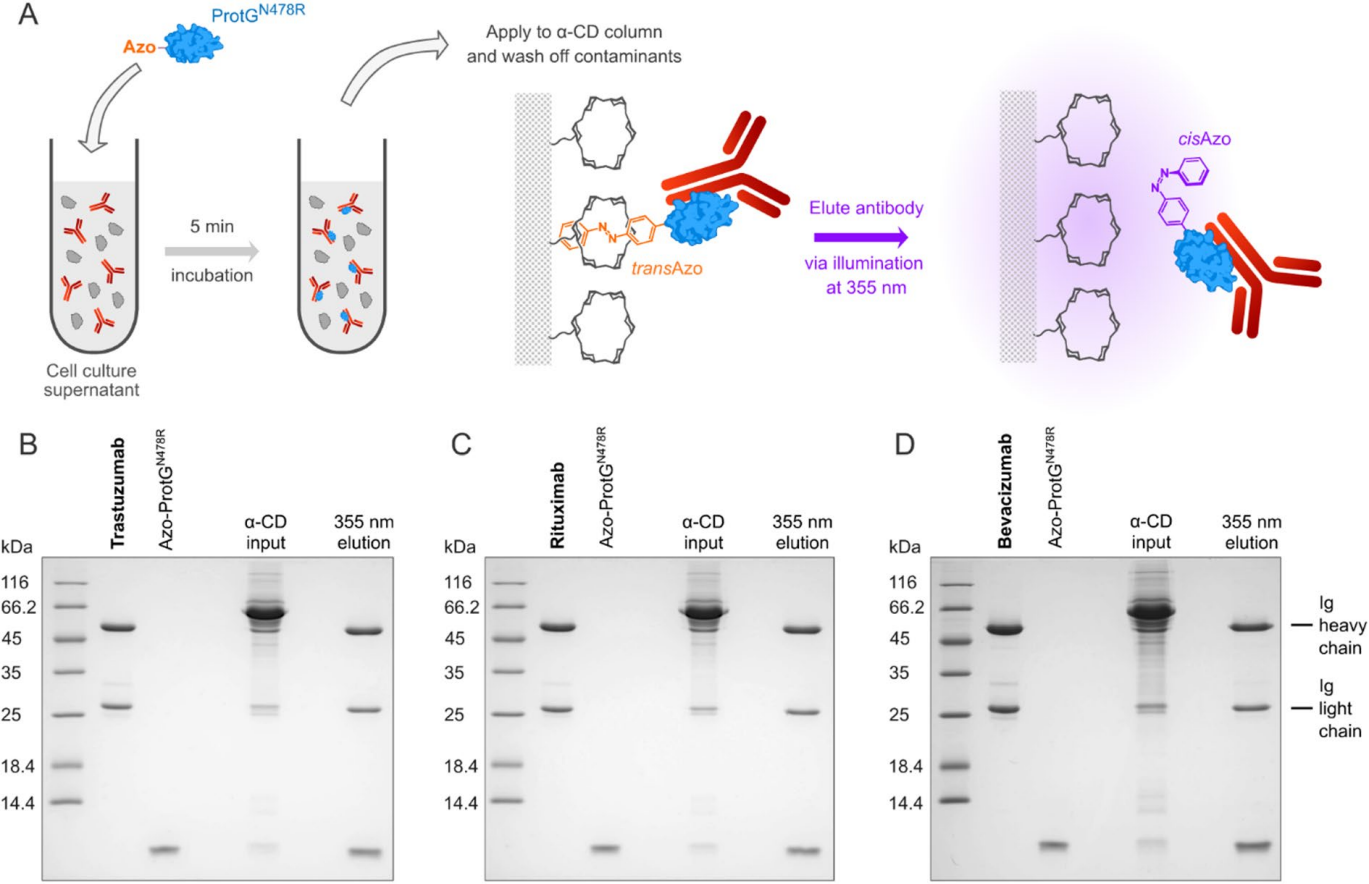
Light-controlled affinity-purification of antibodies using the Azo-ProtG^N478R^ adapter molecule. (**A**) Scheme of the purification workflow: Azo-ProtG^N478R^ (blue), equipped with the light-responsive Azo-tag (orange), is added to the antibody solution in cell culture medium, and the sample is then loaded onto the α-CD affinity column. The Pap residue of the Azo-tag in its *trans* configuration (under daylight or in the dark) binds specifically to the α-CD groups, thus leading to the retention of the mAb in complex with Azo-ProtG^N478R^ while contaminants are washed off. Upon illumination of the column with mild UV-A light, Pap is switched to the *cis* configuration (purple) and loses its affinity to α-CD, leading to the instant elution of the bound antibody in a physiological buffer. (**B**–**D**) Reducing SDS-PAGE illustrating the efficient one-step purification of antibodies from cell culture media. Trastuzumab (**B**), rituximab (**C**) or bevacizumab (**D**) were applied at 0.2 mg/ml in 2 ml DMEM containing 10% (v/v) FBS and mixed with Azo-ProtG^N478R^ at a molar ratio of ~ 1:4. These samples were loaded onto a 1 ml α-CD column (“α-CD input”), then medium constituents and serum proteins were washed out with Chromatography Buffer (25 mM Tris/Cl pH 8.0, 150 mM NaCl) and, finally, the mAb was eluted in 1.5 ml of the same buffer just via exposure to 355 nm UV-A light (“355 nm elution”).

Taken together, the Asn478 → Arg mutation in ProtG prevents cross-linking of antibodies while preserving high affinity to the Ig Fc region; consequently, Azo-ProtG^N478R^ appeared as an ideal candidate to be used as adapter molecule for the light-controlled affinity purification of mAbs (Fig. 4). This was demonstrated by diluting several medically relevant antibodies to 0.2 mg/ml in DMEM cell culture medium containing 10% (v/v) FBS, thus mimicking a culture supernatant obtained after secretory expression of mAbs in mammalian cells. At this low protein concentration, there were no prominent bands visible for the heavy and light Ig chains in Coomassie-stained SDS-PAGE compared with the strong background of serum proteins, in particular bovine serum albumin (BSA).

Subsequently, purified Azo-ProtG^N478R^ was added at an approximately 4:1 ratio to each sample, i.e. in a moderate molar excess over the antibody (now considering the presence of only two symmetry-related binding sites per mAb). After incubation for 5 min, the clear solution was applied to an α-CD affinity column in a shaded laboratory. While the Azo-tag in its *trans* configuration led to the specific binding of the mAb complex to the α-CD matrix, the medium and all contaminants were quickly washed off, as demonstrated previously^7^. However, upon illumination of the column with mild UV-A light using 355 nm wavelength LEDs, the Azo-tag switched to the *cis*-state, and the antibody instantly eluted within 1.5 column volumes in a mild buffer at physiological pH (Fig. 4).

In this manner, trastuzumab, bevacizumab and rituximab were quantitatively isolated from the culture medium under native conditions and all recovered in a highly pure state without detectable contaminations. Notably, while a lower antibody concentration was chosen so far to illustrate the high specificity of the light-controlled affinity purification using Azo-ProtG^N478R^, the actual binding capacity of the α-CD affinity column is much higher, with ≥ 3 mg bound mAb per ml bed volume (see Methods). As Azo-ProtG^N478R^ binds to the IgG Fc region at the junction between the C_H_2 and C_H_3 domains, which is remote both from the antigen-binding sites and from the interfaces with Fcγ receptors and FcRn, the resulting antibody solution—obtained in a buffer of choice—is directly suitable for immunochemical or cell culture assays. However, in cases where the complex between the antibody and the adapter molecule may interfere with further investigations, for example during in vivo studies, we have confirmed that Azo-ProtG^N478R^ can be easily removed from the mAb preparation by gel filtration in the presence of a low concentration of urea (Supplementary Fig. S3)—similarly as demonstrated before with a different Ig-binding protein^20^.

## Discussion

The cloning and application of bacterial Ig-binding proteins, starting with protein A from *Staphylococcus aureus*^1^, followed by protein G from group G *Streptococci*^2^ and protein L from *Peptostreptococcus magnus alias Finegoldia magna*^21^, has revolutionized the affinity purification of antibodies both in an academic setting and in the biopharmaceutical industry. All three proteins are multi-domain surface proteins anchored in the cell wall of the pathogenic Gram-positive host bacteria, where they promote evasion from the humoral immune response^22,23^. Apparently, one of their functions is to decorate the bacterial cell surface with plasma proteins from the infected host, which is mediated by mutually homologous domains that exert specific binding activity towards immunoglobulins or albumin.

In the context of antibody research and technology, the Ig-specific domains of these bacterial binding proteins are of primary interest. While most of the commercially available protein A affinity chromatography matrices today employ several repeat units derived from its B or C domains^24,25^, various isolated Ig-binding domains are in use for research purposes: for example, the engineered "Z-domain" derived from domain B of protein A^26^, the C2 domain of protein G^27^ and one of the repeated B domains of protein L^4^. Interestingly, in each protein A, G and L the repeated copies of the Ig-binding domains are mutually homologous, however their three-dimensional structures differ between the individual bacterial surface receptors: the Ig-binding domains of protein A exhibit a three-helix fold (59 residues)^28^ whereas those of protein G comprise just one α-helix which is positioned diagonally across a four-stranded β-sheet (56 residues)^12^. Although the B domain of protein L (76 residues) binds to the Ig light chain instead of the heavy chain it shares the same fold with the Ig-binding domains of protein G (see Fig. 1E) and exhibits a similar binding mechanism^29^.

The Ig-binding domains of protein A and G are mainly applied for their affinity towards the Fc regions of IgG subclasses from various species, whereas the one of protein L is known to be directed against the V_κ_ domain in the light chain, which occurs in the context of different Ig classes (including IgA, IgD, IgE and IgM). Notably, all of the three types of Ig-binding domains have dual binding activities (see Fig. 1A, B and C). Protein A binds to the IgG Fc region^28,30^ with its α-helices 1 and 2 but it can also bind—less strongly—to the Fab portion^31^ of many antibodies via its α-helices 2 and 3. Protein G, on the other hand, binds to the IgG Fc predominantly via the exposed side of its α-helix, interestingly at the same elbow region as protein A^20^, while it can also bind to the Fab—again, less strongly—via the tip of its α-helix and the lower edge of its β-sheet^27,31^. Importantly, the binding sites of both bacterial receptor proteins within the heavy chain of the Fab differ: protein A interacts with the V_H_ domain, but protein G binds to the C_H_1 domain. In contrast, protein L has two binding sites—with high and low affinity, respectively—for the V_κ_ domain within the light chain, i.e. on the other side of the Fab. The first interaction arises from one edge of its β-sheet and part of the α-helix while the secondary binding site is almost symmetrically arranged on the other side of its β-sheet, also involving the α-helix, and targets the same surface region on the V_κ_ domain of a second Fab^29^.

The biological role of these recurrent dual binding activities towards antibodies remains obscure, also in the light of the clustering of multiple Ig-binding domains within each bacterial Ig-receptor. Possibly, this feature may contribute to the broad Ig isotype and species cross-reactivity or it may function as a kind of superantigen to eliminate immune cells via crosslinking of B cell receptors. Generally, this kind of polyvalency should result in a strong avidity effect which, in fact, could be beneficial for purposes of affinity separation, such as the conventional protein A/G/L affinity chromatography of antibodies, or of immunoprecipitation. However, the situation is different when the application involves the defined formation of a soluble 1:1 (or 1:2) complex between an antibody and a bacterial Ig-binding protein in solution as it is the case, for example, when using ProtG as an adapter molecule for the light-controlled affinity chromatography of mAbs. Even though the affinity of a single ProtG domain towards the Fab region of an IgG1 antibody is much lower than for the Fc portion, with K_D_ values of 20 µM versus 500 nM, this dual binding activity is sufficient to provoke a strong precipitation effect on the mAb if mixed at equivalent ratio, as demonstrated here. Obviously, this phenomenon benefits from the circumstance that the two binding sites of ProtG towards Fab and Fc do not sterically overlap (see Fig. 1B) and that this bivalent/bispecific binding activity occurs in the context of multiple interactions and synergistic avidity effects as part of a larger immune complex, as explained above (see Fig. 2B).

Indeed, the low but practically relevant binding activity of ProtG towards Fab has been utilized for the affinity purification of this class of antibody fragments^13^. Furthermore, attempts were made to improve this secondary binding activity of the ProtG domain by protein engineering^27^. Otherwise, not much attention has been paid to the dual binding activity of this and the other bacterial Ig-binding domains and, to our knowledge, there were no previously published efforts to render protein G monovalent. Considering the somewhat persistent multiple Ig-binding properties within this entire protein family it is surprising that a single amino acid exchange implemented here in ProtG, Asn478 → Arg, not only effectively destroys measurable affinity towards the Fab but also completely abolishes its precipitation activity when mixed with a mAb. Thus, the resulting mutant Azo-ProtG^N478R^, which can be expressed at high yield in *E. coli* and retains full binding activity towards the Ig Fc portion, provides a viable adapter reagent for the convenient and mild one-step isolation of mAbs from cell culture supernatants via Excitography. While this innovative light-controlled purification technique so far has been established for the laboratory scale, and application for industrial manufacturing of mAbs likely needs further engineering, the beneficial monovalent binding properties of the ProtG^N478R^ mutant developed here should also be useful in other areas of antibody research.

## Materials and methods

### Antibodies

The following antibodies were used in this study: trastuzumab (Herceptin®; Roche, Grenzach-Wyhlen, Germany), bevacizumab (Oyavas®, STADA Arzneimittel, Bad Vilbel, Germany) and rituximab (Rixathon®; Sandoz, Kundl, Austria). The Fab fragment of trastuzumab^32^ was obtained as a functional recombinant protein via periplasmic secretion in *E. coli* as previously described^18^. The IgG1 Fc fragment was obtained by proteolytic cleavage of bevacizumab^33^ with recombinant *S. pyogenes* IdeS protease^34^ carrying a C-terminal His_6_-tag, produced in the cytoplasm of *E. coli*. 2 mg of the antibody was mixed with 60 µg IdeS in 1 ml phosphate-buffered saline (PBS) and incubated at room temperature for 4 h. After complete digest, as checked by SDS-PAGE, the protein solution was applied to a MabSelect protein A column (Cytiva, Freiburg, Germany), washed with PBS (pH 7.4) to remove the F(ab)_2_ fragment as well as the protease, and the Fc fragment was eluted with 0.1 M glycine/HCl pH 2.0. The eluate containing the pure protein was immediately neutralized with 1 M Tris and dialyzed against PBS.

### Expression and purification of recombinant protein G

Expression and purification of the Azo-tagged ProtG variants were performed as described^7^. Briefly, the C2 domain of *streptococcal* protein G with the N-terminal Azo-tag (H_2_N-Gly-Pap-Gly-Pro-) and the C-terminal *Strep*-tag II was expressed from the pSB19-PapRS#34 plasmid using amber stop codon suppression (TAG encoding the Pap residue). The protein was produced in *E. coli* NEBExpress(lowRF1) in 1 L LB/Amp medium supplemented with 0.2 mM Pap and 0.8 mM hydroxypropyl-β-cyclodextrin as well as 0.2% (w/v) arabinose and 0.5 mM isopropyl-β-D-thiogalactopyranoside (IPTG) to induce expression of the engineered aminoacyl-tRNA synthetase and ProtG, respectively. The culture was further incubated under agitation for 18 h at 30 °C. Then, the bacteria were harvested by centrifugation, mechanically disrupted in SA buffer (100 mM Tris/Cl pH 8.0, 150 mM NaCl), and the cleared lysate was subjected to StrepTactin affinity chromatography (IBA Lifesciences, Göttingen, Germany). Azo-ProtG was eluted with SA buffer containing 2.5 mM D-desthiobiotin and the peak fractions were subjected to Excitography. To this end, the sample was applied onto an α-CD affinity column with 1 ml bed volume in a shaded laboratory. After washing with 3 ml Chromatography Buffer (25 mM Tris/Cl pH 8.0, 150 mM NaCl), the protein was eluted in the same buffer by illuminating the column with LEDs at 355 nm UV light. The N478R mutation was introduced into the pSB19-PapRS#34-ProtG expression plasmid via QuikChange mutagenesis (Agilent, Santa Clara, CA) and the resulting Azo-ProtG^N478R^ mutant was expressed and purified as above. Purified proteins were analyzed by electrospray ionization mass spectrometry (ESI–MS) in the positive ion mode using an impact II instrument (Bruker Daltonics, Bremen, Germany).

### Light-controlled antibody purification

0.4 mg antibody from the commercial vial was mixed with 2 ml Dulbecco’s Modified Eagle’s Medium (DMEM; PAN-Biotech, Aidenbach, Germany), supplemented with 10% (v/v) fetal bovine serum (FBS; PAN-Biotech). 45 µl of a 250 µM stock solution of the purified Azo-ProtG^N478R^ was added and, after gentle mixing, the solution was incubated for 5 min at room temperature. The sample was applied to a 1 ml α-CD affinity column in a shaded laboratory. After washing with 2.5 ml Chromatography Buffer, the antibody in complex with Azo-ProtG^N478R^ was eluted with 1.5 ml of the same buffer via illumination at 355 nm with LED UV light. To estimate the column capacity for antibody purification, up to 5 mg bevacizumab was mixed with Azo-ProtG^N478R^, at 1:2 molar ratio, in 2 ml Chromatography Buffer, and the light-controlled affinity chromatography was performed as above. To remove Azo-ProtG^N478R^ from the purified antibody complex, 1.5 M urea was added from a 8 M stock solution in Chromatography Buffer and, after 15 min incubation, size exclusion chromatography (SEC) was performed on a Superdex 200 10/300 GL column (Cytiva) using 1.5 M urea in Chromatography Buffer.

### Turbidity assay

150 µl of the solution of an antibody or antibody fragment, diluted in Chromatography Buffer, was mixed with an equal volume of an Azo-ProtG solution to reach a final concentration of 0.1 mg/ml or 0.5 mg/ml for the antibody and a molar ratio mAb:Azo-ProtG = 1:1–1:50. Samples were incubated at room temperature for the period indicated, resuspended by repeated pipetting, and transferred to a 500 µl quartz cuvette with 10 mm path length (108.002-QS; Hellma, Müllheim, Germany). The optical density at 400 nm (OD_400_) was measured in an Ultrospec 2100 pro spectrophotometer (Amersham Biosciences, Amersham, UK). To account for the small absorbance in this wavelength region due to the Pap side chain, measured values were corrected for the OD_400_ determined for a corresponding solution of Azo-ProtG alone.

### Dynamic light scattering (DLS)

40 µl solutions, in Chromatography Buffer, of trastuzumab (1 mg/ml, 6.9 µM) and of each Azo-ProtG variant (28 µM), or buffer alone, were filtered through Ultrafree 0.22 µM centrifugal filters (Merck Millipore, Burlington, MA). After mixing and 20 h incubation at room temperature, samples were transferred to a fluorescence quartz cuvette with 3 × 3 mm^2^ path lengths (105.251-QS, 8.5 mm center height; Hellma). The macromolecular size distribution was determined in a Zetasizer Nano-S photometer (Malvern Instruments, Malvern, UK) using the backscatter mode (173° angle).

### SPR measurements

Real-time surface plasmon resonance (SPR) spectroscopy was performed on a BIAcore X100 instrument (GE Healthcare, Munich, Germany) with HBS/T (20 mM HEPES/NaOH pH 7.4, 150 mM NaCl, 0.005% (v/v) Tween 20) as flow buffer. A 5 µg/ml dilution of trastuzumab, Fc fragment, or recombinant Fab in 10 mM Na-acetate pH 5.0 was immobilized onto a CM5 sensor chip (Cytiva) using amine coupling chemistry to achieve a surface density of ΔRU = 1000–1500. A reference flow channel was equally incubated with coupling reagents but quenched without addition of protein; response signals measured for the reference channel were subtracted from the flow channel harboring the immobilized protein. For the Fc and the mAb, Azo-ProtG variants were injected in a 3:1 dilution series (7290–10 nM), whereas for the Fab fragment a 2:1 dilution series was applied (64–1 µM for wild-type Azo-ProtG, 256–16 µM for the N478R mutant). For all runs, buffer was injected as blank and corresponding signals were subtracted from the measured sensorgrams prior to analysis^35^. K_D_ values were calculated from multi-cycle measurements via a steady-state fit for a 1:1 binding model using BIAcore X100 evaluation software version 2.0.

### Circular dichroism spectroscopy

CD spectroscopy was performed on a J-1500 spectropolarimeter (Jasco, Groß-Umstadt, Germany) equipped with a 1 mm path length quartz cuvette (110-QS; Hellma) using Spectra Manager 2.15 software (Jasco). Spectra were recorded at 20 °C from 190 to 250 nm by accumulating 8 runs (bandwidth 1 nm, scan speed 100 nm/min) using a 0.15 mg/ml protein solution in CD buffer (50 mM K_2_SO_4_, 20 mM K-P_i_ pH 7.5). To determine melting curves, the ellipticity at 217 nm was recorded for 0.3 mg/ml protein solutions in CD buffer from 20 to 95 °C, every 0.1 °C step, using a temperature increase of 1 K/min. The data were fitted using an equation for a two-state unfolding transition und subsequently normalized to the fraction of protein unfolded^36^.

## Supplementary Information

Below is the link to the electronic supplementary material.

Supplementary Material 1

## Data Availability

The biochemical data that support the findings of this study are provided within the manuscript or the supplementary information file. All crystal structures discussed in this work are available from the Research Collaboratory for Structural Bioinformatics Protein Data Bank, RCSB PDB (https://www.rcsb.org).
