# Supplementary material for: Engineering of an Fc-specific monovalent protein G for the light-controlled affinity purification of antibodies
Source: Sci Rep. 2025 Oct 31;15:38111. doi: 10.1038/s41598-025-25894-5 (PMC12579241; doi:10.1038/s41598-025-25894-5)
Supplement: Supplementary file 1 — Supplementary Material 1 [file 41598_2025_25894_MOESM1_ESM.pdf]

Supplementary Information

Engineering of an Fc-specific monovalent protein G for the light-controlled affinity purification of antibodies

Peter Mayrhofer & Arne Skerra

Chair of Biological Chemistry, School of Life Sciences, Technical University of Munich, 85354 Freising, Germany

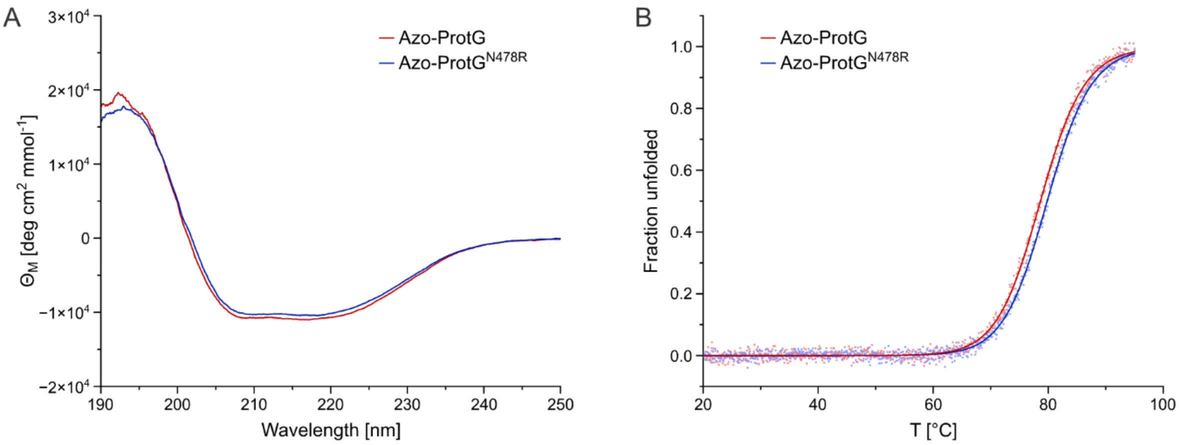

Supplementary Figure S1. Determination of the folding stability of Azo-ProtG by circular dichroism (CD) spectroscopy. (A) Overlay of the CD spectra of Azo-wtProtG and Azo-ProtG^{N478R}. (B) Melting curves of the wild-type and mutated Azo-ProtG versions determined as change in ellipticity at 217 nm. Data points were normalized as fraction of unfolded protein (light red and light blue dots, respectively). The fitted unfolding curves are displayed as solid lines, resulting in T_M values of 78.7 °C and 79.8 °C, respectively.

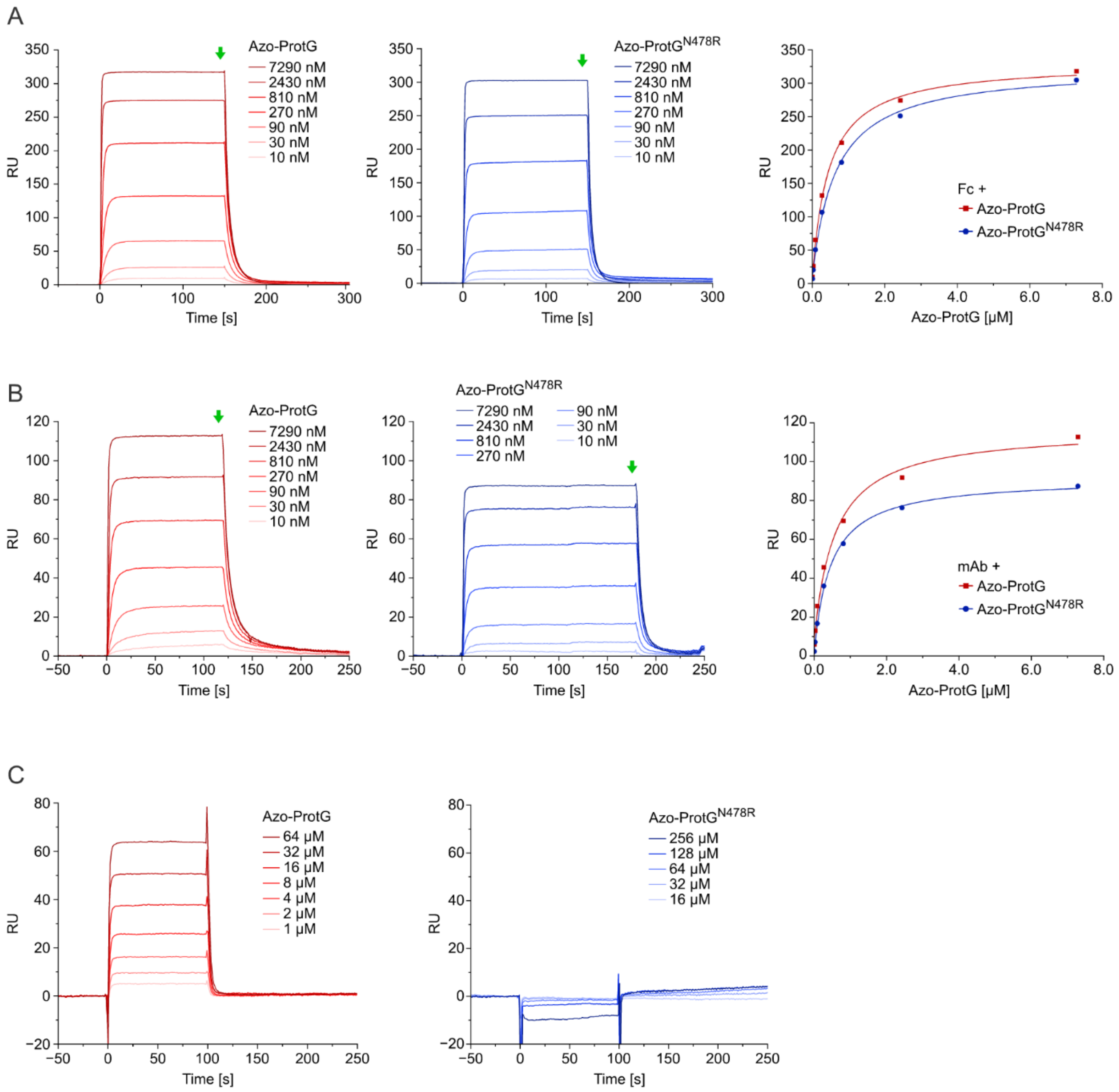

Supplementary Figure S2. Exemplary sensorgrams (raw data) of SPR multi-cycle measurements and subsequent data fitting for the interaction between Azo-ProtG versions and different Ig proteins. (A) Response signals of Azo-ProtG (red, left) or Azo-ProtG^{N478R} (blue, middle) applied as analytes in concentration series on a chip with the immobilized Fc fragment. Signals measured at the end of the contact time (marked with a green arrow) were subjected to a steady state fit using the Biacore software (right), resulting in the K_D values reported in Table 1. (B) Same analytes and concentration series as in (A), measured on a chip with immobilized trastuzumab. (C) Sensorgrams obtained for a chip with the immobilized trastuzumab Fab. While binding was detectable for Azo-wtProtG (red, left), no positive SPR signal was measurable for Azo-ProtG^{N478R} (blue, right; an observed decrease in signal response was probably due to the high sample concentration, up to 256 µM).

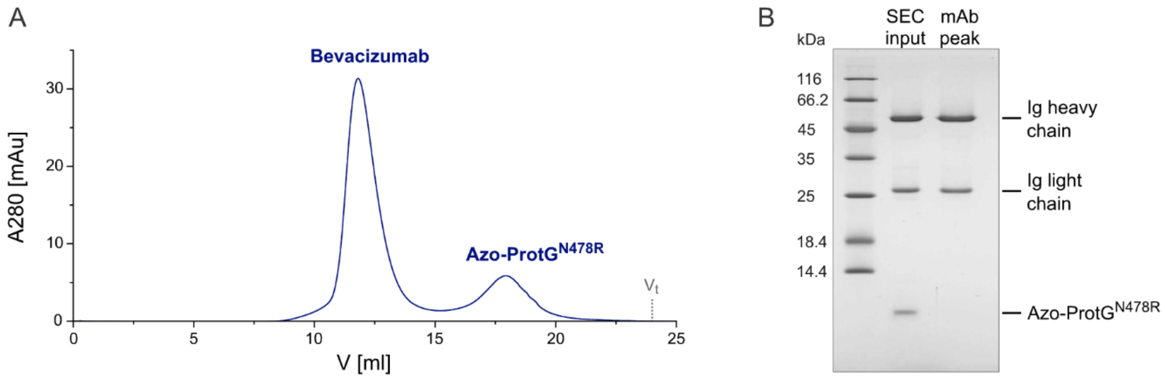

Supplementary Figure S3. Depletion of Azo-ProtG^{N478R} from the bevacizumab complex, after the light-controlled affinity purification, via size exclusion chromatography (SEC) in the presence of a low concentration of urea. (A) SEC profile of a Superdex 200 10/300 GL run, performed with Chromatography Buffer containing 1.5 M urea. (B) The sample eluted in the first peak only contained the pure antibody (mAb).
